# Association between dynamic changes in lumbar back temperature during professional driver training and lumbar muscle endurance: a longitudinal study

**DOI:** 10.3389/fphys.2026.1803242

**Published:** 2026-08-03

**Authors:** Xiaolong Sang, Li Yi, Yuhan Tian, Zhibo Wang, Chaoyue Sun, Xin Wang, Wei Gu

**Affiliations:** 1 Department of Traditional Chinese Medicine, Naval Medical University, Shanghai, China; 2 Naval Medical Center, Shanghai, China

**Keywords:** back temperature, driver, infrared thermography, longitudinal study, lower back pain, lumbar muscle endurance, occupational health

## Abstract

**Objective:**

To investigate the dynamic changes in lumbar-back temperature associated with low back pain (LBP) during the training period of professional drivers and its influencing factors, analyze the correlations of infrared thermal imaging parameters with lumbar muscle endurance and LBP symptoms.

**Methods:**

A longitudinal follow-up study was conducted involving 135 male truck driving trainees from a driving school. The follow-up period spanned four months, with four assessment points (T1-T4). Ultimately, 132 trainees (18–30 years; mean 22.6 ± 2.2 years) completed the full follow-up (effective rate: 97.8%). Infrared thermography was employed to measure mean lumbar-dorsal temperature, temperature gradients, Cumulative Heat Alteration (CHA) and Jumpwise Unevenness Estimation (JUE) in the lower back. Concurrently, lumbar muscle endurance tests and physical measurements were conducted, while low back pain symptoms and functional impact were assessed using the Numerical Rating Scale (NRS) and Oswestry Disability Index (ODI). Data were analyzed using repeated measures ANOVA, Pearson/Spearman correlation analysis, and multivariate linear regression, with a significance level of α=0.05.

**Results:**

During training, the average lumbar-dorsal temperature initially increased gradually, then stabilized, before showing a marked decrease in the final phase; The lumbar temperature gradient continued to expand, with both group and individual temperature heterogeneity significantly increasing by the training’s conclusion (P<0.001). Correlation analysis revealed significant positive correlations between lumbar muscle endurance test duration and both CHA scores (R = 0.2, P = 0.02) and JUE scores (R = 0.25, P = 0.004). After adjusting for confounding factors such as Body Mass Index (BMI), regular physical exercise and muscle percentage, lumbar muscle endurance was the only statistically significant independent factor affecting CHA (P = 0.033) and JUE (P = 0.015). BMI showed a marginal correlation (0.05<P<0.2), and other variables had no independent statistical association.

**Conclusion:**

During professional driver training, characteristic dynamic changes occur in lumbar-back temperature, and these changes are independently associated with lower back muscle endurance. These metrics provide a non-invasive reference for exploring the relationship between lumbar muscle function and LBP. Targeted lumbar endurance training combined with appropriate BMI management can support lumbar health protection for professional drivers and some sedentary populations.

## 1 Introduction

Low back pain (LBP) is a common clinical symptom rather than an independent disease, referring to pain, stiffness, or discomfort occurring in the region between the lower margin of the twelfth rib and the gluteal cleft (the gluteal fold). It may or may not be accompanied by radiating pain in one or both lower limbs (Hartvigsen et al., 2018). The occurrence of LBP among professional drivers is closely associated with occupational exposure factors, including microtrauma to lumbar tissues caused by whole-body vibration during driving and sustained muscle tension resulting from continuous operation (Lu, 2020). Studies confirm that the 12-month prevalence of LBP among professional drivers globally reaches 53%-56%, significantly higher than that in the general population (15%-20%), with a persistent upward trend in the incidence of chronic low back pain (duration ≥3 months) (Joseph et al., 2020). Such pain not only leads to lumbar dysfunction and reduced quality of life among drivers (Garg et al., 2020), but may also induce distractions, sudden pain episodes, and consequent driving errors, thereby increasing traffic accident risks.

Existing research predominantly employs cross-sectional designs. Joseph et al. (2020) systematic review of 19 relevant studies revealed only one cohort study, with the remainder being cross-sectional. Such designs can only reveal static associations at a single point in time, failing to capture the dynamic progression of low back pain or clarify the temporal effects of risk factors such as cumulative driving duration (Jia et al., 2024). Concurrently, current assessment systems over-rely on subjective scales such as the Visual Analogue Scale (VAS), the Numerical Rating Scale (NRS) and the Oswestry Disability Index (ODI), yielding results influenced by individual pain tolerance and emotional state, with coefficient of variation reaching 15%-20% (Garg et al., 2020). Furthermore, 80%-90% of LBP cases are “non-specific”, lacking objective biomarkers reflecting tissue physiological dysfunction, resulting in inadequate accuracy for early risk screening and disease monitoring (Zheng et al., 2025).

Infrared thermography, as a non-invasive, non-contact detection method, quantifies surface temperature distribution based on infrared radiation principles. By measuring surface temperatures in the lumbar region, it indirectly reflects local blood circulation, inflammatory responses, and muscle metabolic abnormalities. Its findings correlate significantly with lumbar muscle function and pain intensity (Albuquerque et al., 2025). It has been preliminarily applied in pain assessment for patients with chronic non-specific low back pain and military pilots (Mastalerz et al., 2022; Ramirez-GarciaLuna et al., 2022), but no systematic longitudinal follow-up studies have yet been conducted in professional drivers, a high-risk group for low back pain, precluding clarification of the temporal association between thermal imaging parameters and the evolution of low back pain symptoms. Consequently, this study aims to investigate the dynamic association between subjective LBP symptoms and lumbar thermal imaging parameters in professional drivers through four follow-up assessments conducted between July and October. It seeks to identify key factors influencing these temperature changes, thereby providing evidence-based support for the precise prevention and management of lower back pain in professional drivers.

## 2 Study population and methods

### 2.1 Study population

This study selected 135 male trainees enrolled in truck driving training at a driving school between July and October 2025. The research protocol was approved by the Ethics Committee of Shanghai Changhai Hospital (Approval No.: CHEC2023-191), all participants were fully informed about the study and voluntarily signed a written informed consent form; the study was conducted in strict accordance with the ethical requirements of the Declaration of Helsinki throughout. Inclusion criteria: ① Driving experience ≥ 1 year, with certain driving experience; ② Age ≥ 18 years; ③ Voluntary participation in the study with willingness to complete four follow-up data collection sessions; ④ No history of serious organic lumbar spine conditions (such as a history of surgery for lumbar disc herniation, lumbar fractures or lumbar tumors); no history of serious skin conditions or infections of the lower back (which may affect the results of thermal imaging); no history of neuropathic pain, rheumatoid arthritis, reactive arthritis or other conditions that may interfere with the assessment of low back pain; ⑤ Not overweight (BMI < 30) or obese, and metabolically healthy; ⑥ During the follow-up period, no medication that might affect the planned study results, such as antidepressants or painkillers, was taken; ⑦ Full comprehension of the study content and voluntary signing of the informed consent form. Exclusion criteria: ① Participants unable to complete all four data collection sessions during follow-up due to injury, illness, resignation, etc.; ② Participants with concomitant severe diseases of major organs (heart, lungs, kidneys, etc.) or malignant tumors; ③ Participants with a history of acute lumbar trauma or surgery within the past three months; ④ Conditions such as skin diseases and infections of the lower back that may affect the results of thermal imaging examinations of the lower back; ⑤ Participants with key study data missing at a rate exceeding 10%.

### 2.2 Research methodology

This study employed a prospective longitudinal follow-up design. The initial baseline assessment was completed during the first month of enrollment (T1), followed by monthly follow-up assessments (T2, T3, T4) over a four-month period. During the study period, one participant withdrew and completed only the T1 assessment, while two participants commenced employment early and completed only the first three follow-up assessments. Ultimately, 132 participants completed all four follow-up assessments, yielding an effective follow-up rate of 97.8%.

### 2.3 Research tools and assessment content

**Figure 1:**
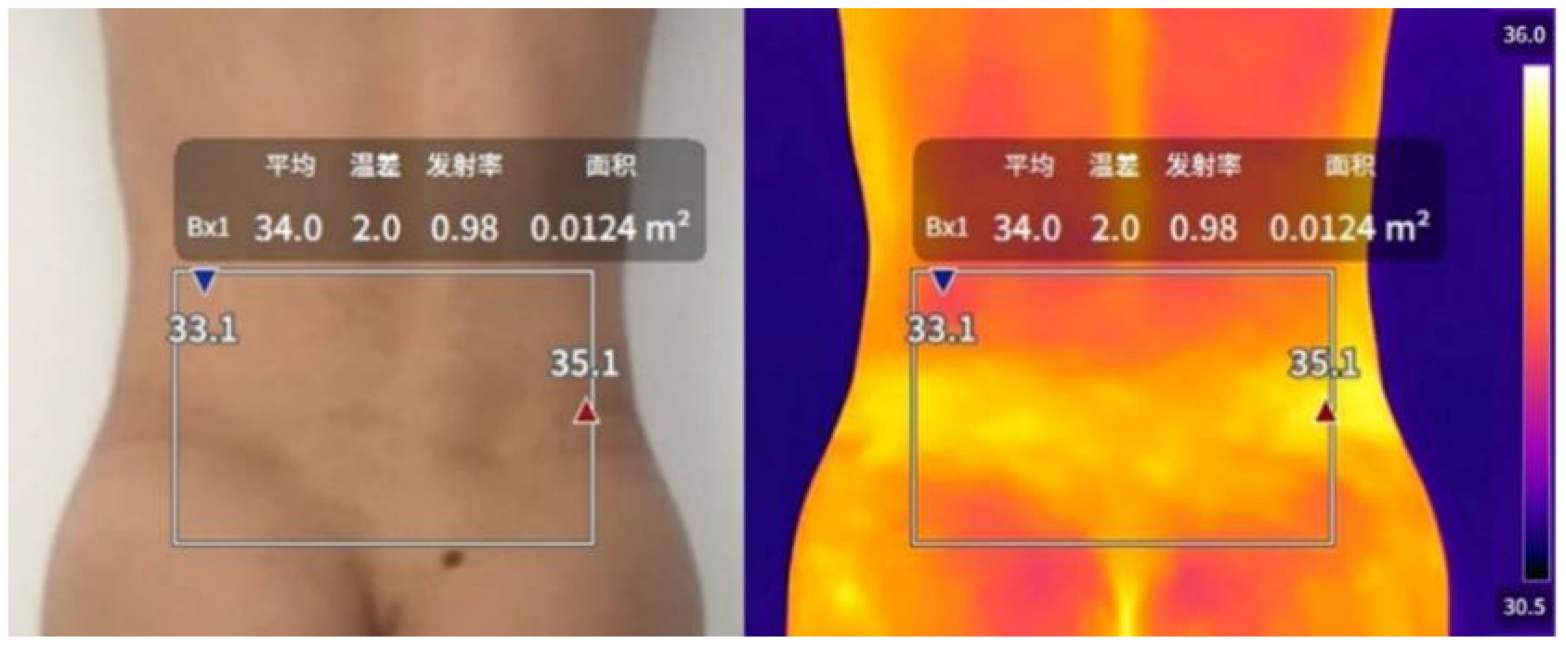
Target area of the lumbar and dorsal regions highlighted in the thermal imaging scan.


1. Collection of Basic Subject Information: A customized Driver Lower Back Pain Status Questionnaire was used to gather relevant information, including: demographic background, medical history (particularly lumbar spine-related conditions), and characteristics of back pain (covering core aspects such as onset time, frequency of episodes, duration, triggering factors, relief methods, and pain nature).
2. Pain and Functional Impairment Assessment: Two standardized scales were employed to evaluate pain and functional impairment, both focusing on subjects’ low back condition during the preceding week: ① Numerical Rating Scale (NRS) for Pain: Scores range from 0 (no pain) to 10 (worst possible pain). Participants selected their score based on perceived intensity to assess low back pain severity. ② Oswestry Disability Index (ODI) Questionnaire: This 45-point questionnaire covers dimensions related to daily living activities. Higher scores indicate greater limitations in daily functioning due to low back pain, assessing the level of functional impairment caused by the pain.
3. Physical and Functional Testing: All physical and functional assessments were conducted by uniformly trained personnel following standardized protocols, with the specific procedures as follows: ① Physical Measurements: Body weight and body fat percentage were determined using a standard body fat scale. Height, waist and hip circumferences were measured with a soft tape measure, and calculate the Body Mass Index (BMI) and waist-to-hip ratio. ② Core Muscle Function Test (Graded Plank): Participants assume a prone position, supporting themselves with elbows and forearms directly beneath the chest, legs shoulder-width apart, and toes as the other support point. The body is raised and maintained in a straight line parallel to the ground. This is graded from 1 to 8 levels of difficulty, progressing from easier to more challenging. ③ Lumbar Muscle Endurance Test: Subjects lie prone with hands clasped behind the head. Using the anterior superior iliac spine as the dividing line, they lift the upper body as high as possible and maintain this position. The maximum duration sustained is recorded as the lumbar muscle endurance assessment metric.
4. Lumbar-Back Temperature Data Acquisition (Thermal Imaging Technology): Data collection utilizes an infrared thermal imager (Model: Fotric 348L) under strictly standardized environmental conditions: room temperature maintained at 20–24 °C, relative humidity at 50–60%. Prior to acquisition, subjects fully exposed their skin surface and rested for 5 minutes to acclimatize. Simultaneous visible light and thermal imaging dual-view scans of the lumbar-dorsal region were then captured (as illustrated in the left and right examples). During image analysis, the target lumbar-dorsal region was delineated within the thermal image (**Figure 1**: Target area in the lower back selected within the thermal image), and two types of core data are extracted: basic temperature parameters and longitudinal composite dynamic indicators: ① Basic temperature parameters: the average temperature of the target detection area, and the temperature difference between the highest and lowest temperatures within the target area; ② Longitudinal composite dynamic indicators: constructed using average temperature data from the lower back and lumbar region at four follow-up time points: T1 (baseline at enrollment), T2 (1 month of training), T3 (2 months of training) and T4 (3 months of training). The specific definitions and calculation formulas are as follows: Cumulative Heat Alteration (CHA): Used to quantify the net change in lumbar and dorsal temperature from baseline to the end of the follow-up period. The calculation formula is:

$$CHA=|T_{T4}-T_{T1}|$$

In the equation, TT1 represents the overall average temperature of the lumbar and dorsal regions at the T1 baseline, and TT4 represents the overall average temperature of the lumbar and dorsal regions at the T4 follow-up endpoint; Taking the absolute value eliminates interference from temperature fluctuations in either direction, focusing solely on the net change from baseline to endpoint; Jumpwise Unevenness Estimation (JUE): used to quantify the extent of dynamic fluctuations in lumbar and dorsal temperature throughout the entire follow-up period, calculated as:


$$JUE=|T_{T2}-T_{T1}|+|T_{T3}-T_{T2}|+|T_{T4}-T_{T3}|$$

In the formula, TT1–TT4 represent the overall average temperatures of the lower back at the four follow-up time points T1–T4; by summing the absolute values of temperature changes between adjacent time points, the temperature fluctuations across the entire cycle are fully captured, with higher values indicating poorer dynamic stability of the lower back temperature. The region-of-interest selection and parameter extraction for all images were performed independently by two specialist physicians using a blinded method. The consistency of continuous temperature data was verified using Cohen’s Kappa coefficient (k > 0.85), ensuring the reliability of the test results.

### 2.4 Statistical methods

Data were analyzed using SPSS 26.0 software with a significance level of α=0.05. ① Descriptive statistics: Continuous variables were expressed as “mean ± standard deviation” or “median (interquartile range)” according to distribution type; categorical variables were presented as “frequency (percentage)”. ② Time Trend Analysis: Repeated measures ANOVA compared differences in NRS scores, ODI indices, and thermal imaging parameters (mean temperature, temperature difference) across the four time points (T1–T4); pairwise comparisons used LSD method; ③ Correlation Analysis: Pearson correlation for normally distributed variables and Spearman’s rank correlation for non-normally distributed variables assessed associations with low back pain-related indicators; ④ Multiple regression analysis: Using CHA and JUE respectively as the dependent variables, variables with P < 0.10 in univariate analysis (including lumbar muscle endurance, BMI, exercise habits, low back pain symptoms and body composition indicators, etc.) were included. Models were constructed using stepwise regression, and the models’ coefficient of determination (R²), adjusted R², and the unstandardized regression coefficients (B) for each variable, along with their 95% confidence intervals and corresponding P-values, were reported.

## 3 Results

### 3.1 Basic characteristics and baseline data of study participants

This study initially enrolled 135 male truck driving trainees newly enrolled at a driving school between July and October 2025, employing a prospective longitudinal follow-up design (with four follow-up time points: T1-T4). During the study period, one trainee withdrew after completing only the T1 assessment, and two trainees completed only the first three follow-up assessments due to early employment. Ultimately, 132 trainees (Age range: 18–30 years; mean age: 22.6 ± 2.2 years) completed all four follow-ups, yielding an effective follow-up rate of 97.8%. Among the 132 male trainees who completed the full follow-up: Ethnic composition was predominantly Han Chinese (123 participants, 93.2%), with nine participants (6.8%) from other ethnic groups, Prior to training, the average daily driving duration was 1.6 ± 1.3 hours. Physical measurements revealed an average height of 174.5 ± 5.3 cm, average weight of 69.1 ± 8.2 kg, and average BMI of 22.7 ± 2.1 kg/m². Regarding physical exercise habits, 107 participants (81.1%) engaged in regular exercise (≥3 sessions per week, ≥30 minutes per session), while 25 (18.9%) did not. Concerning prior lumbar health, 43 (32.6%) reported a history of lumbar discomfort (non-organic lesions), and 89 (67.4%) had no history of lumbar-related discomfort. All participants had no history of acute lumbar trauma or surgery within the preceding three months and no skin conditions affecting thermal imaging assessment of the lumbar region, thus meeting the inclusion and exclusion criteria for this study.

### 3.2 Characteristics of overall lumbar temperature changes during training

**Figure 2:**
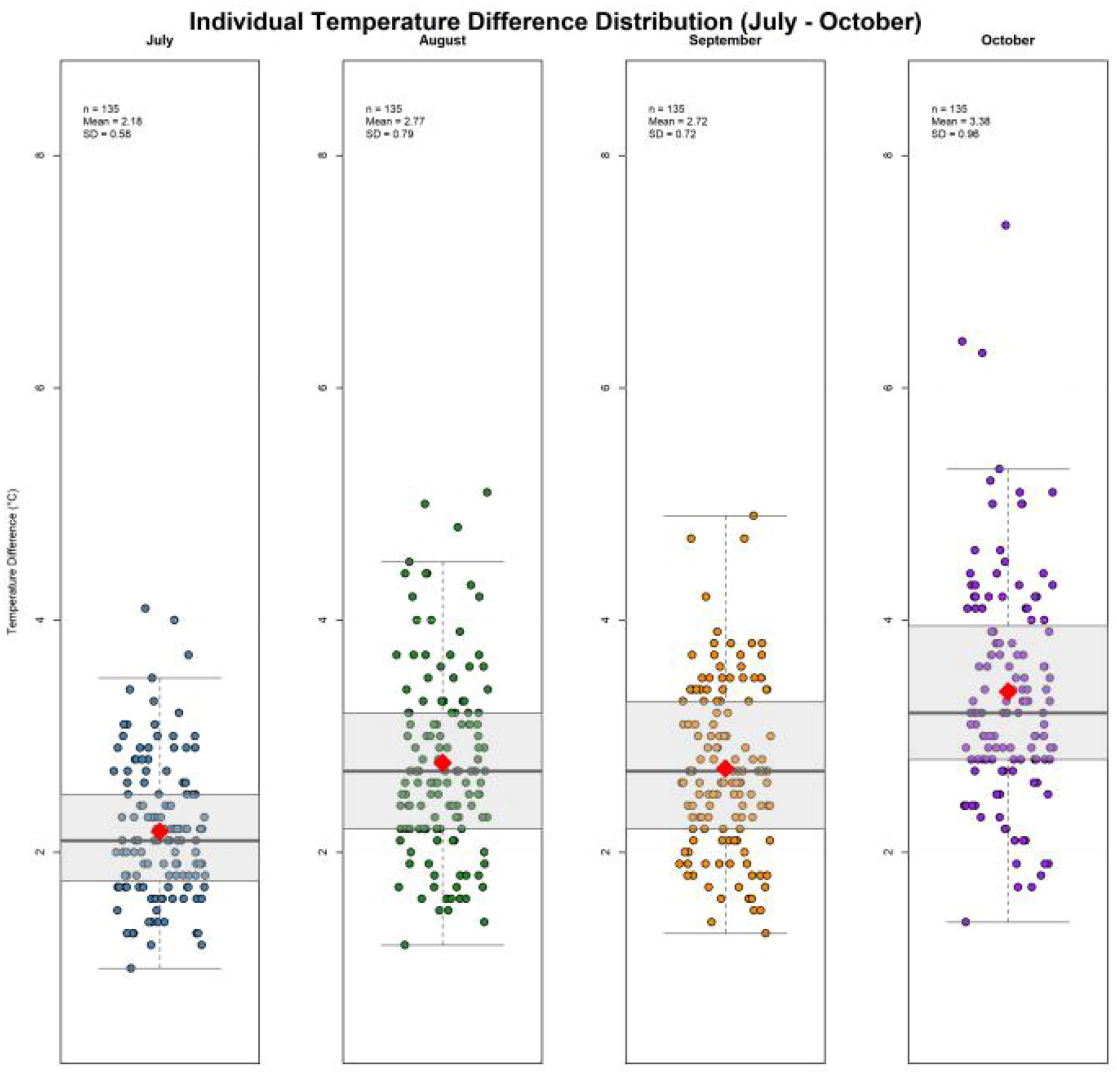
Distribution of temperature differences in the lumbar and dorsal regions of individuals T1 to T4.

**Figure 3:**
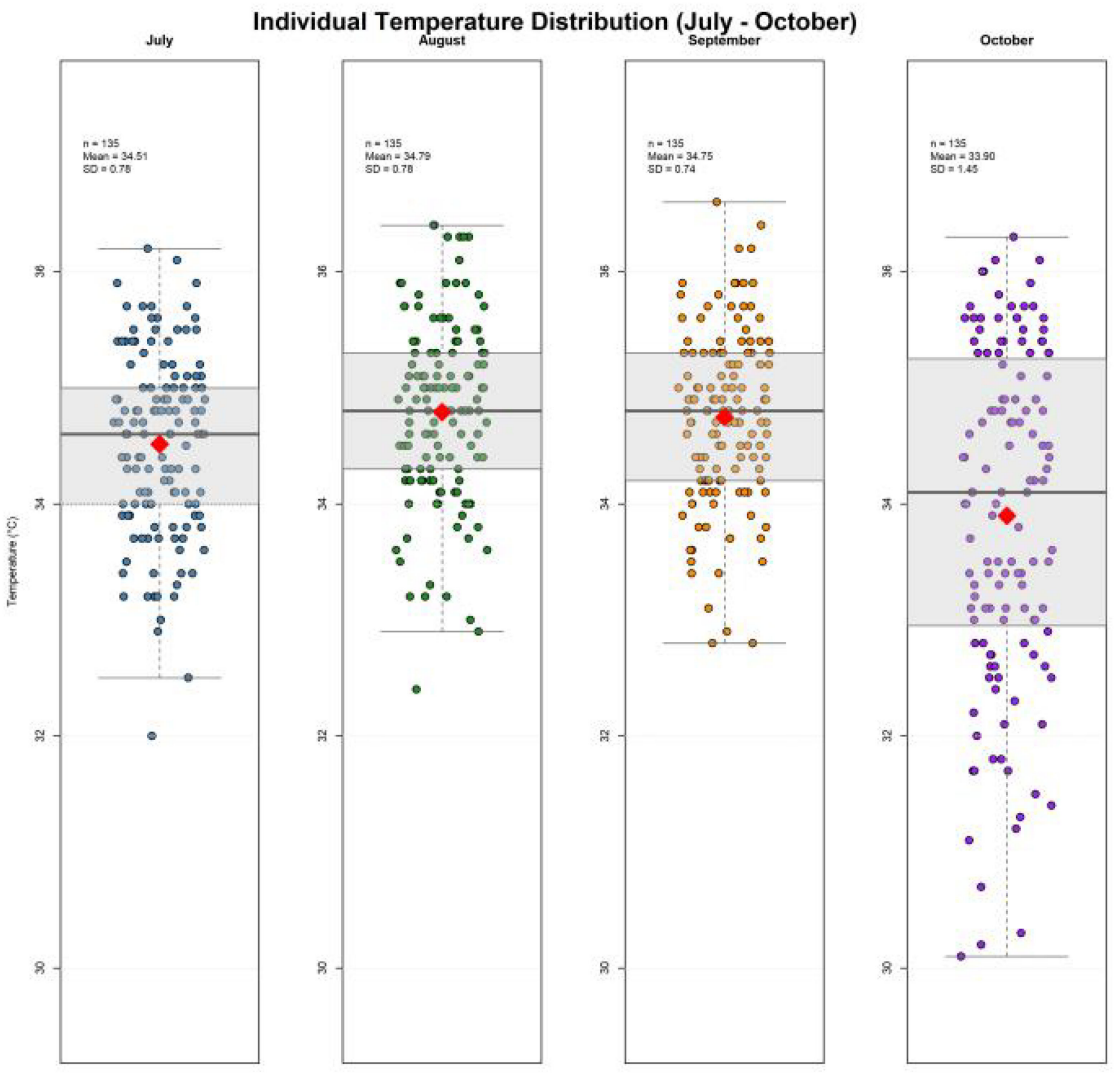
Average temperature distribution across the lumbar and dorsal regions of individuals T1 to T4. This set of images illustrates the distribution of lumbar and back temperature-related metrics across four time points during the training period for 132 research subjects. **Figure 3** depicts the average temperature distribution, while **Figure 2** shows the bilateral temperature difference distribution. Within each sub-image, the jittered scatter plot represents individual data points, the gray area denotes the interquartile range (25th–75th percentile), and the red dot indicates the median for the corresponding time point. The sample size (n) and standard deviation (SD) are labeled above each sub-image.

Statistical analysis of lumbar temperature-related indicators at four time points during the training period for 132 male trainees who completed the full follow-up revealed that the average lumbar temperature exhibited phasic characteristics: the baseline value at T1 was 34.50 ± 0.78 °C, rising to 34.80 ± 0.77 °C at T2, T3 remained at 34.75 ± 0.75 °C, while T4 decreased significantly to 33.90 ± 1.45 °C, with the standard deviation at T4 markedly widening compared to the preceding three time points. Concurrently, the temperature variance reflecting individual dispersion within the cohort progressively increased throughout the training process: from 2.20 ± 0.58 °C at T1, rising sequentially to 2.76 ± 0.79 °C at T2 and 2.72 ± 0.72 °C at T3, before further escalating to 3.38 ± 0.96 °C at T4. The overall differences in mean temperature and temperature variation across the four time points were statistically significant (P<0.001). (**Figure 2**: Distribution of individual lumbar-dorsal temperature differences at T1-T4; **Figure 3**: Distribution of individual lumbar-dorsal mean temperatures at T1-T4).

### 3.3 Correlation analysis between lumbar muscle endurance and lumbosacral temperature stability

#### 3.3.1 Screening of factors influencing lumbar temperature and temperature differential

**Figure 4:**
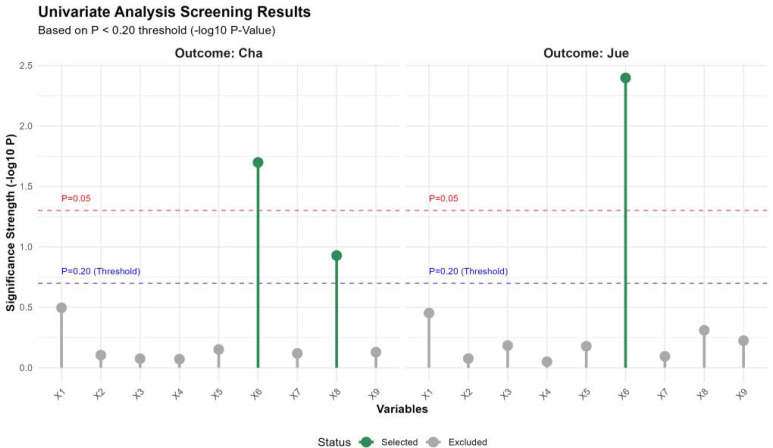
Results of single-factor analysis variable screening. This image presents the significance levels of univariate analyses examining the association between dynamic thermal indicators of the lumbar-dorsal region (CHA, JUE) and related clinical variables with lumbar muscle endurance and lumbar-dorsal injuries. The Y-axis represents significance strength (-log_10_ P-value; higher values indicate stronger association between variable and outcome). The X-axis displays covariates included in the analysis, defined as follows: X1 = Regular physical exercise (≥3 sessions weekly, ≥30 minutes per session), X2 = occurrence of low back pain within the current month, X3 = frequency of low back pain episodes within the past month, X4 = Numerical Rating Scale (NRS), X5 = core muscle function grade (level), X6 = lumbar muscle endurance test duration (s), X7 =Pittsburgh Sleep Quality Index (PSQI), X8 = Body Mass Index (BMI), X9 = muscle mass percentage (%). The red dashed line in the image represents the statistical significance threshold at P = 0.05, while the blue dashed line denotes the marginal significance threshold at P = 0.20. Green dots indicate variables selected for subsequent multivariate regression analysis following univariate screening (including CHA, JUE, and lumbar muscle endurance test time X6), whereas gray dots denote variables failing to meet the screening criteria.

To further clarify the association between dynamic lumbar temperature changes and lumbar muscle endurance, BMI, and injury indicators, we calculated the CHA value (T4-T1) reflecting net temperature change and the JUE value (sum of absolute temperature changes between adjacent time points) reflecting fluctuation amplitude from temperature time series data. These were combined with lumbar temperature parameters (mean temperature, temperature differential) and injury-related indicators (X2: occurrence of low back pain in the current month; X3: frequency of low back pain in the past month; X4: NRS self-rated pain score). Correlation analyses were conducted with the core variables lumbar muscle endurance test time (X6), BMI (X8), and other relevant variables, employing Pearson or Spearman correlation analyses according to the distribution type of the variables. Results indicate that the JUE value, reflecting the amplitude of lumbar-back temperature fluctuations, exhibits significantly stronger correlations with lumbar muscle endurance test time (X6) and lumbar-back injury-related indicators than the CHA value, which reflects net temperature change (see univariate analysis figures).Lumbar muscle endurance test time (X6) was significantly correlated with JUE, lumbar temperature differential and injury indicators (all P<0.05).Conversely, the CHA value demonstrated only weak associations with certain injury indicators, further confirming that fluctuation amplitude provides a more sensitive reflection of muscle functional status. BMI (X8) exhibited moderate correlations (0.05 < P < 0.2) with the aforementioned core outcome measures, though these associations were weaker than those observed for lumbar muscle endurance test duration (X6). This suggests BMI exerts a relatively moderate influence on lumbar temperature dynamics and injury risk, necessitating comprehensive assessment alongside other factors. The correlation results between the remaining variables and outcome measures, combined with the screening outcomes from the univariate analysis (only variables such as JUE, CHA, and lumbar muscle endurance test time (X6) met the inclusion criterion of P < 0.05, while BMI (X8) was at a borderline level of significance), were incorporated into subsequent multiple regression analyses to identify independent influencing factors. (**Figure 4**: Results of univariate variable screening).

#### 3.3.2 Correlation between lumbar muscle endurance and dynamic lumbar temperature indicators (CHA, JUE)

**Figure 5:**
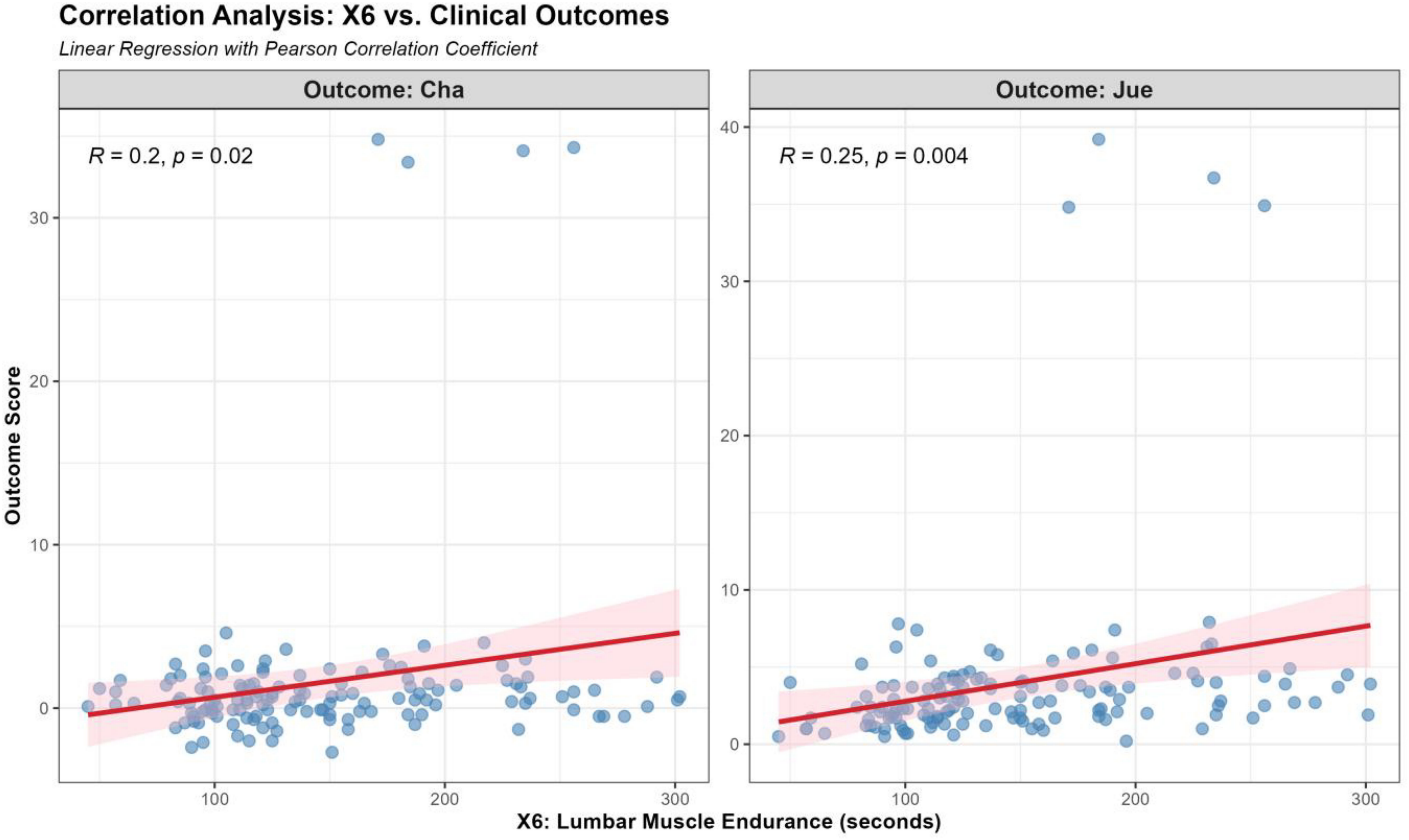
Correlation analysis between lumbar muscle endurance test duration and dynamic indicators of lumbar and back temperature. This image employs Pearson’s correlation test to investigate the relationship between lumbar muscle endurance test duration (X6, horizontal axis, units: seconds) and dynamic indicators of lumbar-dorsal temperature. In the left subplot, the outcome variable is the cumulative heat alteration (CHA) value (vertical axis), defined as the net change in lumbar-dorsal temperature between the fourth and first time points during the training period (T4-T1); The outcome variable in the right-hand subplot is the Jumpwise Unevenness Estimation (JUE) value (y-axis), defined as the sum of absolute temperature changes between consecutive time points during the training period, reflecting the cumulative magnitude of temperature fluctuations. The solid red line represents the linear fit, while the pink region denotes the 95% confidence interval.

Further analysis examined the relationship between lumbar muscle endurance test duration and dynamic lumbar temperature indicators. Pearson correlation tests were employed to investigate associations with net temperature change (CHA, i.e. T_4_ - T_1_) and cumulative fluctuation amplitude (JUE, i.e. the sum of absolute temperature changes between consecutive time points). Results demonstrated a positive correlation between lumbar muscle endurance test duration and CHA values (R = 0.2, 95%CI: 0.004~0.380, P = 0.02), with an even more significant positive association observed for JUE values (R = 0.25, 95%CI: 0.056~0.426, P = 0.004). Both correlations were statistically significant. These findings suggest that greater lumbar muscle endurance (as indicated by longer test duration) correlates with both a larger overall net change in lumbar temperature (CHA) and a higher cumulative amplitude of temperature fluctuations (JUE) during the training period. When considered alongside the phased temperature changes observed during training, this indicates that improvements in lumbar muscle endurance are closely associated with the dynamic adaptation process of lumbar temperature. (**Figure 5**: Correlation analysis between lumbar muscle endurance test duration and dynamic indicators of lumbar temperature).

#### 3.3.3 Multivariate regression analysis of independent factors influencing lumbar-back temperature dynamics

Multivariate linear regression analysis revealed that only lumbar muscle endurance test duration (X6) served as an independent influencing factor across both models examining lumbar-back temperature fluctuation amplitude (JUE) and net temperature change (CHA): within the JUE model, lumbar muscle endurance test duration exhibited a significant positive correlation with JUE values (P = 0.015); while in the CHA model, it also exhibited a significant positive correlation with CHA values (P = 0.033). BMI and other included clinical and behavioral variables showed no independent statistical association after adjusting for confounding factors, indicating lumbar muscle endurance as the core factor influencing dynamic lumbar temperature changes during the training period. (**Table 1**: Multivariate regression analysis of independent influencing factors for lumbar temperature dynamics (JUE, CHA)).

**Table 1:** Multivariate regression analysis of independent factors influencing dynamic temperature indicators (JUE, CHA) in the lumbar and back regions.

Variables	β	B(95%CI)	t	P
JUE				
X1	0.05	0.72(-1.88 to 3.32)	0.548	0.585
X3	0.07	1.29(-1.80 to 4.38)	0.827	0.410
X6	0.22	0.02(0.004 to 0.040)	2.470	0.015
X8	-0.14	0.40 (-1.00 to 0.20)	-1.309	0.193
X9	-0.14	-0.08 (-0.22 to 0.05)	-1.252	0.213
CHA				
X1	0.07	1.00 (-1.51 to 3.51)	0.789	0.431
X6	0.18	0.018 (0.002 to 0.035)	2.154	0.033
X8	-0.13	-0.36 (-0.81 to 0.10)	-1.531	0.128

This presents the results of a multivariate linear regression model with fluctuations in lumbar-dorsal temperature (JUE) and net temperature change (CHA) as dependent variables. Here, β denotes the standardized regression coefficient, B (95% CI) represents the unstandardized regression coefficient with 95% confidence interval, t indicates the test statistic, and P signifies the significance level. The covariates included in the analysis are defined as follows: X1 = regular physical exercise (≥3 sessions per week, ≥30 minutes per session), X3 = frequency of lower back pain episodes in the past month, X6 = lumbar muscle endurance test duration (seconds), X8 = BMI, X9 = muscle mass percentage (%).

## 4 Discussion

### 4.1 Characteristics of lumbar-back temperature variations and their physiological mechanisms

This study conducted statistical analysis on lumbar-back temperature-related indicators during the training period of 132 professional drivers. Results indicate that the average lumbar-back temperature of professional drivers exhibited distinct phased changes throughout training progression: temperatures gradually increased during the initial phase, remained stable during the middle phase, and showed a decreasing trend during the final phase. Concurrently, intra-group temperature variations progressively widened throughout the training program. This pattern indicates a marked increase in lumbar-dorsal temperature heterogeneity at both group and individual levels by the training’s conclusion. This temperature progression aligns closely with the physiological sequence of muscle activation, adaptation, fatigue, and cumulative load during training. During the initial training phase, continuous training stimuli kept the lumbar muscles in an activated state. Increased metabolic heat production during muscle contraction, coupled with accelerated local blood circulation, led to a marked elevation in skin temperature (Vasquez-Bonilla et al., 2025). By the mid-training phase, muscles had progressively adapted to the training load. Metabolic heat production and the body’s heat dissipation processes reached a dynamic equilibrium, stabilizing lumbar and back temperatures. Rodrigues et al. (2025) observed that during this adaptation period, muscle metabolism and regulatory functions became coordinated, with relevant physiological indicators exhibiting steady-state characteristics. By the late training phase, the cumulative effects of prolonged training loads induce microtrauma in muscles, fascial adhesions, and impaired local blood circulation. This leads to reduced muscle heat production coupled with diminished heat dissipation regulation, ultimately manifesting as a decrease in average lumbar and dorsal temperatures. Furthermore, individual variations in muscle fatigue levels and load adaptation capacity exacerbate temperature differentiation within the group, significantly widening the thermal disparity. Regarding quantitative temperature dynamics indicators, the CHA value reflects the overall net temperature change from training initiation to completion, capturing the general trend. Conversely, the JUE value focuses on fluctuation amplitude, sensitively capturing temperature dynamics across training phases. JUE can comprehensively capture temperature fluctuations throughout the entire follow-up period; compared with CHA, which reflects only the net change from baseline to endpoint, it provides a more comprehensive picture of temperature variations. This suggests that JUE values may serve as a preliminary exploratory research tool for investigating lumbar muscle function. This finding further corroborates the close association between temperature fluctuation patterns and muscle physiological function.

### 4.2 Independent associated factor of lumbar muscle endurance in dynamic changes of lumbar-back temperature

Both correlation analysis and multivariate linear regression analysis in this study confirmed that lumbar muscle endurance test duration was the sole independent positive influencing factor for dynamic lumbar-back temperature indicators (CHA, JUE). It exhibited significant positive correlations with CHA (R = 0.2, 95%CI: 0.004~0.380, P = 0.02) and JUE (R = 0.25, 95%CI: 0.056~0.426, P = 0.004). This association remained statistically significant after adjusting for confounding factors including BMI, physical exercise, and muscle mass percentage. None of the other variables included in the analysis demonstrated an independent association. Notably, this effect is relatively small, which is understandable given that lumbar skin temperature is regulated by multiple interacting factors, including systemic metabolism, autonomic nervous function, and environmental exposure. But as the only statistically significant, modifiable independent predictor found in our study, it provides a valuable actionable target for lumbar health protection in high-risk professional driver populations. These results indicate that the greater the back muscle endurance of the participants, the greater the net change in back temperature and the greater the overall range of dynamic fluctuations observed during the follow-up period. Furthermore, lumbar muscle endurance was significantly associated with injury-related indicators such as the onset of low back pain, its frequency, and NRS scores. At the first follow-up, 43 participants (32.6%) reported a history of lower back discomfort (non-organic in nature); at the final follow-up, 61 participants (46.2%) reported such a history. This represents a cumulative increase of 18 new cases (an incidence rate of 13.6%). Furthermore, as lumbar muscle endurance declined, the overall incidence of lower back pain showed an upward trend. From a physiological perspective, as the core stabilizing muscle group of the lumbar region, the endurance of the lumbar muscles is related to the biomechanics of the lower back and local blood circulation (Frizziero et al., 2021). The greater the endurance of the lumbar muscles, the better the biomechanical stability of the lower back; this can effectively reduce non-physiological stretching and contraction of the muscles during training and occupational activities, thereby alleviating pain and reducing the likelihood of lower back injuries (Chen and Wang, 2025). Mechanistically, greater lumbar muscle endurance enables more pronounced metabolic adaptation across training phases, resulting in larger cumulative temperature fluctuations. This reflects adaptive physiological variation, not clinical functional instability. Considering the occupational characteristics of professional drivers, this group remains in prolonged sedentary positions, subjecting lumbar muscles to sustained static loads. This predisposes them to chronic muscle tension, circulatory impairment, and cumulative fatigue (Davarian et al., 2012). The independent association with lumbar muscle endurance confirmed by this study highlights the specific value of conducting research on lumbar muscle endurance among professional drivers. Conversely, BMI exhibits only marginal correlations (0.05 < P < 0.2) with the aforementioned temperature and injury-related indicators. Its influence on lumbar physiological states is fundamentally indirect: Firstly, it may indirectly affect local blood circulation and thermal regulation by altering static load distribution in the lumbar region and increasing abdominal fat accumulation, which compresses vasculature and nerves in the lumbosacral area. However, this indirect effect is often overshadowed by the dominant influence of core muscle function (such as lumbar muscle endurance) (Bayartai et al., 2023). Secondly, the high homogeneity of the professional driver cohort in this study resulted in minimal overall variability for such indicators, making it challenging to demonstrate significant correlations in statistical testing.

### 4.3 Clinical and practical value of research on lumbar and back health protection for professionally sedentary populations

This study, focusing on professional drivers, systematically elucidates the physiological patterns of dynamic lumbar-back temperature changes during training periods and the independent association effect of lumbar muscle endurance. Given the occupational health reality of high incidence rates of chronic lumbar-back injuries among professional drivers and sedentary occupations (Jia et al., 2024), The findings of this study provide a preliminary basis for the management of lower back health in this population. Firstly, the exploratory analysis in this study found that dynamic indicators of lower back temperature can provide a non-invasive and convenient basis for research into the functional status of the lumbar muscles; specifically, the temperature fluctuation amplitude indicator JUE is more closely associated with lumbar muscle endurance than the CHA value, which reflects overall net changes. Furthermore, skin temperature monitoring, with its simplicity, non-invasiveness, and rapid on-site implementation, overcomes the limitations of traditional muscle function assessments that rely on specialized equipment and are difficult to deploy at scale in occupational settings. It provides a potential exploratory tool for lumbar muscle function assessment in occupational populations, whose clinical utility for LBP risk identification requires further prospective validation (Hergenroeder et al., 2022). Secondly, strengthening the core muscles could serve as a potential intervention strategy for improving lower back function in professional drivers (Guo et al., 2025), and may provide guidance for research into interventions aimed at promoting lumbar and back health in the working population. Standardized endurance training enhances core lumbar muscle function, thereby counteracting the root causes of prolonged sitting—accumulated static lumbar loads and circulatory impairment. Furthermore, this study focuses on non-invasive monitoring of lumbar temperature during professional driver training, thereby enriching the body of evidence regarding the physiological dynamics of the lumbar region in this population. Its methodology and conclusions can be extended to certain sedentary occupations such as office workers and long-haul flight attendants, providing a reference paradigm for establishing occupational health protection systems for lumbar health across all sedentary professions.

Although this study systematically examined the relationship between dynamic changes in the lower back during the training period of professional drivers and lumbar muscle endurance, certain limitations remain that require further refinement in subsequent research. Firstly, all 132 professional drivers included in this study were male, with no female drivers included, indicating a clear gender selection bias. Given the differences between men and women in terms of muscle mass, lumbar physiological structure and the tolerance of lumbar and back muscle loads, the conclusions of this study can only reflect the physiological characteristics of male professional drivers. Future studies should increase the sample size of female participants to improve the generalizability of the findings; Secondly, this study focused solely on a short-term longitudinal follow-up during a four-month driver training period. It did not conduct long-term follow-ups on changes in lumbar and back temperature or lumbar muscle endurance after the training period, when drivers commenced actual work duties. Consequently, it was unable to establish a causal relationship between physiological changes during the training period and long-term occupational lumbar injuries. The study period should be extended to include long-term follow-ups to clarify the association between short-term physiological changes and long-term occupational health; Thirdly, this study has only revealed a statistical association between lumbar muscle endurance and temperature dynamics, but lacks specific interventions targeting lumbar muscle endurance. Future research should increase the sample size and conduct relevant randomized controlled trials (RCTs), such as interventions to improve lumbar muscle endurance through core muscle function training or traditional Chinese exercise methods, to further validate the causal relationship and physiological mechanisms between the two; Fourthly, although this study strictly standardized the immediate ambient temperature and humidity for each imaging session, cumulative seasonal physiological adaptation across the four-month follow-up was not statistically adjusted. The temperature decrease observed at T4 (October) is consistent with both cumulative muscle fatigue and seasonal thermoregulatory adjustment, and these two explanations cannot be disentangled in the current analysis. This limitation specifically affects the interpretation of CHA; Fifth, the test-retest reliability and MDC for CHA and JUE have not yet been established, which limits the scope of interpretation of inter-subject differences in correlation and regression analyses.

## 5 Conclusions

Through tracking the dynamic changes in lumbar and back temperature during the training period of professional drivers, this study observed a three-stage evolutionary trend: initial rise, subsequent stabilization, and eventual divergence. Multivariate statistical analysis further confirmed that lumbar muscle endurance is independently associated with these dynamic temperature changes, while BMI demonstrated only a marginal correlation. This finding suggests that targeted lumbar muscle endurance training, combined with appropriate BMI management, may effectively alleviate muscle fatigue and reduce the risk of lower back injuries, offering a practical approach to protecting the lower back health of professional drivers and certain groups of people who spend long periods sitting. Subsequent research should first establish the test-retest reliability of CHA and JUE, then further elucidate the physiological mechanisms underlying lumbar temperature dynamics through larger sample sizes and regionalized precision thermography, while exploring more targeted intervention strategies.

## Data Availability

The original contributions presented in the study are included in the article/supplementary material. Further inquiries can be directed to the corresponding authors.
